# Extended dissipative truncated predictive control strategy for conic-type delayed networked control systems affected by cyber attacks and sensor distortion

**DOI:** 10.1038/s41598-025-30817-5

**Published:** 2025-12-06

**Authors:** T. Narenshakthi, S. Dharani

**Affiliations:** Department of Mathematics, School of Advanced Sciences, Vellore Institute of Technology, Vellore, 632014 Tamil Nadu India

**Keywords:** Conic-type non-linearity, NCSs, Extended dissipative, Sensor distortion, Cyber attacks, Engineering, Mathematics and computing

## Abstract

This paper investigates a truncated predictive control (TPC) strategy for delayed conic-type nonlinear networked control systems (CNNCSs). The proposed framework addresses key issues such as sensor distortion, system uncertainty, extended dissipativity performance (EDP), and cyber-attacks. Specifically, cyber-attacks are modeled using a Bernoulli distribution, while sensor distortion is represented by a Markov jump system (MJS) model. To handle these challenges, lyapunov stability theory (LST) and linear matrix inequalities (LMIs) are employed to establish sufficient conditions that guarantee global stability of the networked control systems (NCSs). The TPC approach effectively manages the combined effects of nonlinearities, time delays, and network imperfections. By constructing a suitable Lyapunov–Krasovskii functional (LKF) and applying appropriate inequality techniques, the conditions are formulated in an LMI framework. The derived results ensure that CNNCSs maintain desired performance levels under adverse conditions. Finally, two numerical examples are presented to demonstrate the validity and effectiveness of the proposed method.

## Introduction

The concept of NCSs was introduced in the 1970s, driven by advancements in control technology, computer technology, and network communication. Since the 21st century, NCSs have drawn attention and advanced significantly. Signal transmission between many locations is crucial in many practical systems because it is frequently challenging to have the plant, controller, sensor, and actuator all in one place. These elements are frequently connected via network media in contemporary industrial systems, resulting in the creation of so-called NCSs^1,2^.

In NCSs, parametric uncertainty and external disturbances pose a significant challenge, making it crucial to address the both. It is particularly difficult to achieve an exact implemented controller that satisfies the needs in real-time operations. Because errors or uncertainties may emerge during the development of controllers. Time delays in control systems can manifest in various forms, such as constant delays, time-varying delays, and infinitely distributed delays^3^. In many systems, including NCSs, chemical processing, aircraft, underwater exploration, mechanical and electrical engineering, biology, and especially remote control systems, time delays are a common occurrence. This study specifically addresses time-varying delay among the three forms^4–6^.

Many earlier studies were based on the assumption of accurate sensors; however, in practical applications, sensor distortion is common and therefore should not be ignored. In Ref^7^., sensor distortion was modeled by a Bernoulli distribution. However, assuming a Bernoulli distribution neglects the dependence between sensor distortion occurrences over time. To further address this issue, sensor distortion can be more accurately modeled using approaches such as the MJSs. MJSs have been extensively researched because they may describe systems whose parameters or structures may exhibit abrupt changes, which illustrates many real-world issues with an efficient solution^8^. For instance^9^, models network time delay as a Markov process.

Nonlinearities are unavoidable in practice due to disturbances, modeling errors, and limitations in analysis. Nonlinearities remain a key focus in control theory, as many engineering nonlinearities can be represented by forms such as conic-type, Lipschitz, locally sinusoidal, or dead-zone nonlinearities, for instance^10,11^. Conic-type nonlinear systems have recently gained significant attention as a special class of systems, where the nonlinearity is confined within an *n*-dimensional hypersphere defined by two linear systems determining its radius and center. Studying conic-type nonlinear NCSs is therefore of practical relevance^12^.

Network security research has gained popularity in the last few decades. Malevolent adversaries may alter or steal data while it is being transmitted, which could lead to a decline in system performance or even a loss of control as the communication networks are publicly accessible. To enhance the security of the system, cyber-attacks are taken into consideration. Replay attacks, denial-of-service attacks (DoSA) and deception attacks (DA) are the three typical attacks that have been identified and thoroughly examined for NCSs^13^. The DoSA is to disrupt or stop normal network traffic by overwhelming the servers, infrastructure, and services in the targeted systems^14^. DA involves the attacker interfering with the system’s regular data using the restricted tampering model, then injecting the tampered data to reduce system performance and undermine stability^15^. In Ref^16^., the combination of DoSA with DA is considered for nonlinear NCSs under dynamic event-triggered fault-tolerant control. The network has been enhanced with a few new features, which take the shape depicted in Refs^17–22^..

External disturbances can significantly degrade the performance of control systems. To address this, the concept of EDP was proposed in Ref^23^. as a means to enhance system stability under such conditions. The EDP encompasses special cases such as $H_{\infty }, L_2-L_{\infty },$ passivity and $(\mathcal {Q},\mathcal {S},\mathcal {R})$-dissipativity. The idea of EDP has supported the analysis of stability in various systems, including NCSs that are mean-square asymptotically stable (MSAS) as shown in Ref^23^., and networked singular systems in Ref^24^..

Stability analysis of systems with input delay is a central issue in control theory. To manage such delays, prediction-based feedback strategies are introduced to estimate system states across the delay interval for control purposes. Classical methods like the Smith predictor^25^, finite spectrum assignment^26^, and model reduction^27^have contributed foundational solutions. In particular, model reduction through state transformation using a finite integral over the input history allows the original delayed system to be represented in a delay-free form. When disturbances are present, this transformation is adapted to support control design for linear time-invariant systems, as shown in Ref^28^.. However, despite their conceptual simplicity, predictor-based approaches often involve distributed terms in the feedback law, making them difficult to implement in practice. To avoid the infinite-dimensionality of feedback laws, a finite-dimensional predictor-based method, referred to as the TPC, is introduced.

Various control strategies have been explored for NCSs affected by time delays and cyber attacks. However, TPC for NCSs is still an open problem. In Ref^29^., TPC is formulated for delayed semi-Markovian jump systems. In Ref^30^., the TPC is investigated for interconnected time-varying delayed non-linear systems, extending further to Lipschitz nonlinear systems in Ref^11^. . Furthermore, in Ref^31^., reinforcement learning-based tracking control is investigated for NCSs under DoSA. In Ref^32^., truncated predictor feedback effectively compensates for long time-varying input delays. Prior works studied uncertainty and disturbance estimator (UDE) control for time-varying systems^33^. Although several studies have explored dissipative control and delay-dependent stability in NCS, the combined influence of sensor distortion, system uncertainty, and cyber attacks under a novel TPC has received limited attention. To address this gap, this study develops an extended dissipative control framework for delayed CNNCSs via TPC. In a nutshell, the contributions of our work are outlined below.

1. Contrary to Refs^23,34,35^., the CNNCSs is designed to account for time-varying delays, uncertainty, sensor distortion, and cyber attacks. Simultaneously, sensor distortion is modeled using an MJS.
2. A novel attempt is made to apply the TPC technique to the proposed system to ensure stability, EDP, and compensation for input delay.
3. The LMI is formulated using the LST method, and the corresponding controller gains are derived to ensure both stability and EDP.

The following are the arrangements for the remaining portions of the paper: In Sect. "Problem Statement", the investigation’s core topic is presented, accompanied by essential definitions and lemmas. Stability analysis and EDP are carried out in Sect. "Main results". Section "Numerical example" presents two numerical simulations with illustrative figures, and Sect. "Conclusion" concludes the study.

### Notations

In this paper, $\mathbb {R}^n$ denotes the *n*-dimensional Euclidean space. The mathematical expectation is represented by $\mathbb {E}$. The symbol $\mathbb {R}_+$ represents the set of all positive real numbers. The superscripts $``T'',$ and $``-1''$ indicate the matrix transpose and inverse, respectively. $``I''$ is the identity matrix with appropriate dimension.

**Fig. 1:**
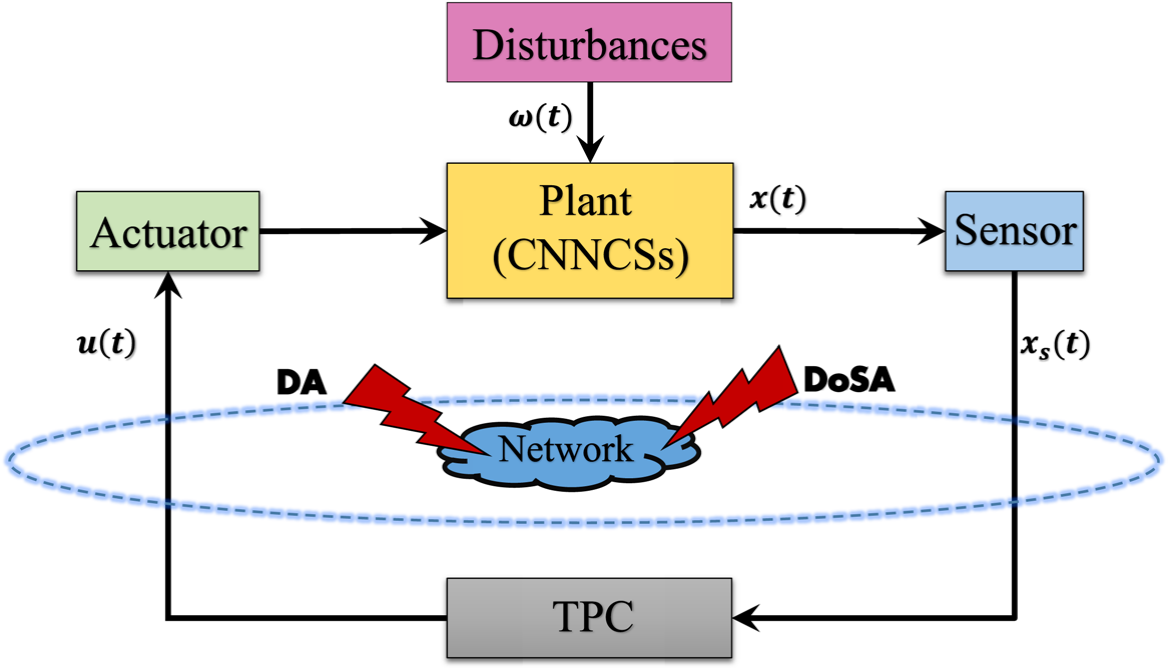
Thematic framework of CNNCSs under sensor distortion and cyber attacks.

## Problem statement

Consider the following nonlinear system with an external disturbance term:

1 $$\begin{aligned} \left\{ \begin{array}{ll} \dot{x}(t)=\mathfrak {g}(x(t),{\omega }(t))+\mathfrak {{N}_2}\mathfrak {u}(\Gamma (t)),\\ y(t)=\mathfrak {{N}_4}x(t), \end{array} \right. \end{aligned}$$

where $x(t)\in \mathbb {R}^n,~\mathfrak {u}(t)\in \mathbb {R}^m,~y(t)\in \mathbb {R}^l,$ and ${\omega }(t) \in L_2[0, \infty )$ are state, control input, measurement output, and external disturbance vectors, respectively. $\Gamma (t):\mathbb {R}_+\rightarrow \mathbb {R}$ is a continuously differentiable function representing time-varying delay, intuitively expressed as $\Gamma (t)=t-\varepsilon (t)$ with $\varepsilon (t)\ge 0$.

### Assumption 1

$\Gamma (t)$ *is a known, continuously differentiable, and invertible function, ensuring* $0<\eta \le \dot{\varepsilon }(t)<\delta$, $\forall ~t\ge 0$. *Given a finite* $\varepsilon _2\ge 0$, $\varepsilon (t)$ *satisfies* $0\le \varepsilon (t)\le {\varepsilon }_2$.

The nonlinearity $\mathfrak {g}(x(t),{\omega }(t))$ lies within the conic sector described below:

2 $$\begin{aligned} \Vert \mathfrak {g}(x(t),{\omega }(t))-[\mathfrak {N_1}x(t)+\mathfrak {N_3}\omega (t)]\Vert \le \Vert [\mathcal {N}_1 x(t)+\mathcal {N}_3 \omega (t)]\Vert . \end{aligned}$$

From (1) and (2), the system can be reformulated as follows:

3 $$\begin{aligned} \left\{ \begin{array}{ll} \dot{x}(t)=\mathfrak {N_1}x(t)+\mathfrak {{N}_2}\mathfrak {u}(\Gamma (t))+\mathfrak {N_3}\omega (t)+\Im (t),\\ y(t)=\mathfrak {{N}_4}x(t), \end{array} \right. \end{aligned}$$

where $\mathfrak {N_\varpi }={N}_\varpi +\Delta {{N}_\varpi (t)}, \varpi =1,2,3,4.$ The matrices ${N}_\varpi , (\varpi =1,2,3,4),~\mathcal {N}_1,$ and $\mathcal {N}_3$ are considered known. The uncertain parameters satisfies the following: $\Delta {{N}_\varpi } (t)= W^{T}\phi (t)\mathcal {J}_{\varpi },~($for$~\varpi =1,2,3,4)$, where $\phi (t)$ is assumed to satisfy $\phi^T(t) \phi (t)\le I$, and the matrices $W,~\mathcal {J}_{\varpi },$ are known. $\Im (x(t),{\omega }(t))=\mathfrak {g}(x(t),{\omega }(t))-[\mathfrak {N_1}x(t)+\mathfrak {N_3}\omega (t)].$ Alternatively, inequality (2) yields the following expression:

4 $$\begin{aligned} \Vert \Im ^T (t)\Vert ^2\le \Vert \mathcal {N}_1 x(t)+\mathcal {N}_3 \omega (t))\Vert ^2. \end{aligned}$$

### Remark 1

Zames^36^ initially introduced the conic-type condition (2), which has since been extensively utilized in modeling nonlinear systems. The function $\Im (x(t),{\omega }(t))$ is typically bounded within an *n*-dimensional hypersphere centered at a linear system $\mathfrak {N_1}x(t)+\mathfrak {N_3}\omega (t)$, with radius $\mathcal {N}_1 x(t)+\mathcal {N}_3 \omega (t)$, which reflects the structure of conic-type nonlinearities.

### Remark 2

Under Assumption 1, the delay $\varepsilon (t)$ is bounded as $0 \le \varepsilon (t) \le \varepsilon _2$. Thus, the prediction time satisfies $0\le \Gamma ^{-1}(t)-t \le {\varepsilon }_2.$

### Sensor distortion

Internal and external factors frequently cause sensor distortion in real-world applications, which results in differences between measured and actual data. This problem is taken into account in this research to make the study more realistic.

This work uses the Markov process to model sensor distortion due to its ability to capture sudden changes. When such distortion occurs,

5 $$\begin{aligned} x_{\mathfrak {s}(t)}=\psi _{\mathfrak {q}(t)}x(t), \end{aligned}$$

where the sensor’s state signal is $x_{\mathfrak {s}(t)}$, and $\psi _{\mathfrak {q}(t)}$ is the distortion parameter with distinct values determined by the Markov process through the random variable $\mathfrak {q}(t)$.

Consider the Markov process $\{\mathfrak {q}(t),t \ge 0\}$, which evolves in continuous time and adopts discrete values from a finite set $\mathbb {V} = \{1,2,...,\mathfrak {a}\}$, uses the following mode transition probabilities:

$$\begin{aligned} {Pr}\{\mathfrak {q}(t+1)=\mathfrak {m}|\mathfrak {q}(t)=\mathfrak {s}\}=\pi _{\mathfrak {s}\mathfrak {m}}, \end{aligned}$$

where $\pi _{\mathfrak {s}\mathfrak {m}}\ge 0,\forall ~\mathfrak {s},\mathfrak {m}\in \mathbb {V}$ and $\sum _{\mathfrak {s}=1}^{\mathfrak {a}}\pi _{\mathfrak {s}\mathfrak {m}}=1.$ Then the Markov process has the following transition probability matrix:

$$\begin{aligned}\Pi =\left[ \begin{array}{cccc} \pi _{11} & \pi _{12} & \cdots & \pi _{1\mathfrak {a}} \\ \pi _{21} & \pi _{22} & \cdots & \pi _{2\mathfrak {a}} \\ \vdots & \vdots & \ddots & \vdots \\ \pi _{\mathfrak {a}1} & \pi _{\mathfrak {a}2} & \cdots & \pi _{\mathfrak {a}\mathfrak {a}} \\ \end{array} \right] . \end{aligned}$$

### TPC design with cyber attacks

Consider the TPC structure as in Ref^30^.:

6 $$\begin{aligned} \mathfrak {\bar{u}}(t)=K e^{\mathfrak {N}_1(\Gamma ^{-1}(t)-t)} x(t), \end{aligned}$$

where the control gain matrix is *K* to be determined. In NCSs, data transmission is vulnerable to malicious interference. This study focuses on two prevalent types of network attacks: DA and DoSA. DA compromise communication integrity by injecting false data $\mathcal {G}(x_\mathfrak {s} (t))$ into the network, effectively replacing the genuine information and disrupting system performance. DA typically arise at random, and this process can be readily described by Bernoulli distribution. It follows that:

7 $$\begin{aligned} \mathfrak {\bar{u}}(t)=K[(1-\alpha (t))e^{\mathfrak {N}_1(\Gamma ^{-1} (t)-t)} x_\mathfrak {s} (t)+\alpha (t) \mathcal {G}(x_\mathfrak {s} (t))], \end{aligned}$$

where $\alpha (t)\in \{0,1\}$, with $\alpha (t)=1$ denoting an active DA and $\alpha (t)=0$ indicating no DA. It is further assumed that $\alpha (t)$ satisfies $\mathbb {E}[\alpha (t)] = \bar{\alpha }$ and $\mathbb {E}[(\alpha (t) - \bar{\alpha })^2] = \bar{\alpha }(1 - \bar{\alpha })$.

#### Assumption 2

*Reference*^37^ *Given a constant matrix* $\mathfrak {S}>0,$ *the cyber attack signal* $\mathcal {G}(x_\mathfrak {s} (t))$ *satisfies*

8 $$\begin{aligned} x^T(t)\mathfrak {S}^T\mathfrak {S}x(t)-\mathcal {G}^T(x_\mathfrak {s}(t))\mathcal {G}(x_\mathfrak {s} (t))\ge 0. \end{aligned}$$

#### Remark 3

Assumption 2 imposes a bounded-gain condition on the cyber attack signal $\mathcal {G}(x_\mathfrak {s} (t))$, ensuring $\Vert \mathcal {G}^T(x_\mathfrak {s}(t))\Vert \le \Vert x^T(t)\mathfrak {S}\Vert$ with $\mathfrak {S}> 0.$ This constraint makes the attacker’s effect tractable in stability analysis. In practice, attackers often proactively limit attack energy to avoid detection by an intrusion detection system.

During transmission, the information may be exposed to interference caused by DoSA. In the absence of DoSA, data is transmitted; under attack, it fails to reach the system. The DoSA indicator $\beta (t)$ equals 0 during an attack and 1 when the system is attack-free. Based on this and (7), we have

9 $$\begin{aligned} \mathfrak {{u}}(t)=\beta (t)\mathfrak {\bar{u}}(t). \end{aligned}$$

#### Remark 4

The $(n+1)$-th DoSA’s, dormant and active periods are denoted by $\mathfrak {D}_{1,n}$ and $\mathfrak {D}_{2,n}$, with lengths $\mathfrak {L}_n$ and $\mathfrak {H}_n$, respectively, where $n \in \mathbb {N}$ is the attack index. Then the duration of each jamming signal is bounded below and upper by the positive scalars $\mathfrak {L}_{n}^{\min },\mathfrak {H}_{n}^{\min }$ and $\mathfrak {L}_{n}^{\max },\mathfrak {H}_{n}^{\max }$ which satisfy $\mathfrak {L}_{n}^{\min }<\mathfrak {L}_{n}< \mathfrak {L}_{n}^{\max },\mathfrak {H}_{n}^{\min }<\mathfrak {H}_{n}< \mathfrak {H}_{n}^{\max }$. Also, $\beta (t)$ satisfies $\mathbb {E}[\beta (t)]=\bar{\beta }.$

From (3) and (9), we have

10 $$\begin{aligned} \left\{ \begin{array}{ll} \dot{x}(t)=\mathfrak {N_1}x(t)+\beta (t)\mathfrak {{N}_2}K[(1-\alpha (t))e^{\mathfrak {N}_1(\Gamma ^{-1} (t)-t)} \psi _{\mathfrak {q}(t)}x(t-\varepsilon (t))+\alpha (t) \mathcal {G}(x_\mathfrak {s} (t))]+\mathfrak {N_3}\omega (t)+\Im (t),\\ y(t)=\mathfrak {{N}_4}x(t). \end{array} \right. \end{aligned}$$

The structure of the formulated problem is depicted in Fig. 1. It provides a clear representation of the system components and their interactions, offering insight into the overall framework.

#### Assumption 3

*Consider matrices* $\Lambda _1, \Lambda _2, \Lambda _3,$ *and* $\Lambda _4$ *under the following conditions*:

1. $$\Lambda _1=\Lambda _1^T \le 0,~\Lambda _3=\Lambda _3^T>0,~\Lambda _4=\Lambda _4^T\ge 0,$$
2. $$(\Vert \Lambda _1\Vert +\Vert \Lambda _2\Vert )\Lambda _4=0.$$

#### Definition 1

Reference^23^ Given matrices $\Lambda _1, \Lambda _2, \Lambda _3,$ and $\Lambda _4$ satisfying Assumption 3, for any $\bar{T}> 0$ and $\omega (t) \in L_2[0,\infty ]$, system (10) is said to be EDP if the following inequality holds:

$$\begin{aligned} \int _{0}^{\bar{T}}\mathfrak {J}(t)dt \ge \sup _{0\le t\le \bar{T}} y^T(t)\Lambda _4 y(t) dt, \end{aligned}$$

where $$\mathfrak {J}(t)=y^T (t)\Lambda _1 y(t)+2y^T(t)\Lambda _2 \omega (t)+\omega ^T(t)\Lambda _3\omega (t).$$

#### Remark 5

The EDP covers four main cases. The following matrices are employed:

$$\mathbf {C_1}.~H_{\infty }: \Lambda _1=-I, \Lambda _2=0, \Lambda _3= \nu ^2 I, \Lambda _4=0;$$

$$\mathbf {C_2}.~L_2 - L_{\infty }: \Lambda _1=0, \Lambda _2=0, \Lambda _3= \nu ^2 I, \Lambda _4=0.09I;$$

$$\mathbf {C_3}$$. Passivity: $$\Lambda _1=0, \Lambda _2=I, \Lambda _3= \nu I, \Lambda _4=0;$$

$$\mathbf {C_4}.~(\mathcal {Q},\mathcal {S},\mathcal {R})$$-dissipativity: $$\Lambda _1=\mathcal {Q}, \Lambda _2=\mathcal {S}, \Lambda _3= \mathcal {R}-\nu ^2 I, \Lambda _4=0.$$

#### Lemma 1

*Reference*^38^ Given $\varphi> 0$ *and an integral function* $J:[u,v] \rightarrow R^n$, *the following inequality holds, if the required integration is adequately defined as*

$$\begin{aligned}\int _{u}^{v} J^T (s) \varphi J(s) ds \ge \frac{1}{v-u} \Bigg (\int _{u}^{v}J(s) ds \Bigg ) ^T \varphi \Bigg (\int _{u}^{v} J(s) ds \Bigg ).\end{aligned}$$

#### Lemma 2

*Reference*^39^ Let $\mathcal {C}$ *be a positive definite matrix. It follows that a scalar* $\sigma \ge 0$ *exists for which the subsequent relation is valid*

$$\begin{aligned} e^{E^T t} \mathcal {C} e^{E t}-e^{\sigma t} \mathcal {C} = -e^{\sigma t} \int _0^t e^{-\sigma \tau } e^{E^T \tau } \mathcal {D} e^{E \tau } d\tau , \end{aligned}$$

*where* $\mathcal {D} = -\mathcal {C}E - E^T\mathcal {C}+ \sigma \mathcal {C}$. *Moreover, if* $\mathcal {D}$ *is positive definite, it follows that*:

$$\begin{aligned} e^{E^T t} \mathcal {C} e^{E t} < e^{\sigma t} \mathcal {C}. \end{aligned}$$

## Main results

In this section, we analyze system (10) with EDP and derive sufficient conditions for its stability, summarized in the following theorem.

### Theorem 1

*Given scalars* $\varepsilon _2,~\xi ,~\mu ,~{z}>0$ *and matrices* $\mathfrak {S}>0,~\Lambda _1~\Lambda _2,~\Lambda _3,~\Lambda _4$. *System* (10) *is MSAS with* $\mathcal {X}=P^{-1}$ *and the controller gain* ${K}=-\mathfrak {N}_2^TP$, *if positive symmetric matrices* $P,Q_1,Q_2$ *exists and meets the following conditions*:

11 $$\begin{aligned} ~\mathfrak {N}_2 \mathfrak {N}_2^T\le \xi \mathcal {X}\end{aligned}$$

12 $$\begin{aligned} \Bigg (\mathfrak {N}_1-\frac{1}{2}\mu I\Bigg )^T+\Bigg (\mathfrak {N}_1-\frac{1}{2}\mu I\Bigg )<0\end{aligned}$$

13 $$\begin{aligned}~[\Theta]_{6\times 6}<0,~[\tilde{\Theta }]_{4\times 4}<0 \end{aligned}$$

where 

$$\Theta _{11}=P\mathfrak {N}_1+\mathfrak {N}_1^TP-2(1-\bar{\alpha })\bar{\beta }P^2 \mathfrak {N}_2^2 \psi _{\mathfrak {q}(t)}+\Pi +(1-\bar{\alpha })^4\bar{\beta }^4 \xi ^2 P^2 \psi ^2_{\mathfrak {q}(t)}$$ $$+(1-\bar{\alpha })^2\bar{\alpha }^2\bar{\beta }^4 \xi ^2 P^2 \psi ^2_{\mathfrak {q} (t)}+2(1-\bar{\alpha })^2\bar{\beta }^2 \xi ^2 P^2 \psi ^2_{\mathfrak {q} (t)}+\bar{\alpha }^2\bar{\beta }^2 \mathfrak {N}_2^4 P^4+5\mathfrak {S}^T\mathfrak {S}+P^2+4\mathcal {N}_1^2+Q_1+Q_2+2\varepsilon _2^2\xi ^2 e^{2\mu \varepsilon _2}\eta ^{-1}$$ $$+2\varepsilon _2^2\xi ^2e^{2\mu \varepsilon _2}\eta ^{-1}\mathfrak {S}\mathfrak {S}^T+2\varepsilon _2^2 e^{\mu \varepsilon _2}\eta ^{-1}\mathcal {N}_1^2-\mathfrak {N}_4\Lambda _1\mathfrak {N}_4^T+P-\mathfrak {N}_4\Lambda _4\mathfrak {N}_4^T,$$ $$\Theta _{13}=2zP\mathfrak {N}_1,\Theta _{15}=2zP\mathfrak {N}_1,\Theta _{16}=P\mathfrak {N}_3+\mathfrak {N}_3^T P+8\mathcal {N}_1\mathcal {N}_3+4\varepsilon _2^2 e^{\mu \varepsilon _2} \eta ^{-1}\mathcal {N}_1\mathcal {N}_3-2zP\mathfrak {N}_1-\mathfrak {N}_4\Lambda _2, \Theta _{22}=-Q_1(1-\delta ),$$ $$\Theta _{33}=-Q_2-2zP^2 (1-\bar{\alpha })\bar{\beta }\mathfrak {N}_2^2 e^{\mathfrak {N}_1 \varepsilon _2}\psi _{\mathfrak {q} (t)}+z^2 P^4\bar{\alpha }^2\bar{\beta }^2\mathfrak {N}_2^4+z^2P^2,\Theta _{35}=-2zP-2zP^2 (1-\bar{\alpha })\bar{\beta }\mathfrak {N}_2^2 e^{\mathfrak {N}_1\varepsilon _2}\psi _{\mathfrak {q} (t)}+z^2 P^4\bar{\alpha }^2\bar{\beta }^2\mathfrak {N}_2^4,$$ $$\Theta _{36}=2zP\mathfrak {N}_3+2zP^2 (1-\bar{\alpha })\bar{\beta }\mathfrak {N}_2^2 e^{\mathfrak {N}_1 \varepsilon _2}\psi _{\mathfrak {q}(t)},\Theta _{44}=-I,\Theta _{55}=-2zP+z^2 P^2,$$ $$\Theta _{56}=2zP\mathfrak {N}_3+2z P,\Theta _{66}=4\mathcal {N}_3^2+2\varepsilon _2^2 e^{\mu \varepsilon _2}\eta ^{-1}\mathfrak {N}_3^2+2\varepsilon _2^2 e^{\mu \varepsilon _2}\eta ^{-1}\bar{N}_3-2zP\mathfrak {N}_3+z^2 P^4\bar{\alpha }^2\bar{\beta }^2\mathfrak {N}_2^4+z^2P^2-\Lambda _3,$$ $$\tilde{\Theta }_{11}=P\mathfrak {N}_1+\mathfrak {N}_1^TP+\Pi +P^2+\mathcal {N}_1^2+Q_1+Q_2-\mathfrak {N}_4\Lambda _1\mathfrak {N}_4^T+P-\mathfrak {N}_4\Lambda _4\mathfrak {N}_4^T,$$ $$\tilde{\Theta }_{14}=P\mathfrak {N}_3+\mathfrak {N}_3^T P+2\mathcal {N}_1 \mathcal {N}_3-2\mathfrak {N}_4\Lambda _2,\tilde{\Theta }_{22}=-Q_1(1-\delta ),\tilde{\Theta }_{33}=-Q_2,\tilde{\Theta }_{44}=-I+\mathcal {N}_3^2.$$

### Proof

This theorem covers two cases.

**Case 1:** Considering the DoSA in sleeping period. Then we construct the LKF as:

14 $$\begin{aligned} \mathcal {V}_1(t)=x^T (t){P}x(t). \end{aligned}$$

Then, applying infinitesimal operator $\mathcal {L}$ and taking expectation along (14) yields

15 $$\begin{aligned} \mathbb {E}\{\mathcal {L}\dot{\mathcal {V}}_1 (t)\}=~&\mathbb {E}\{2x^T(t){P}\dot{x}(t)\}+x^T(t)\sum _{\mathfrak {s}=1}^{\mathfrak {a}}\pi _{\mathfrak {sm}}\Pi x(t)\nonumber \\ =~&x^T(t)P\{\mathfrak {N_1}x(t)+\bar{\beta }\mathfrak {{N}_2}K[(1-\bar{\alpha })e^{\mathfrak {N}_1(\Gamma ^{-1} (t)-t)} \psi _{\mathfrak {q}(t)}x(t-\varepsilon (t))+\bar{\alpha } \mathcal {G}(x_\mathfrak {s} (t))]+\mathfrak {N_3}\omega (t)+\Im (t)\}\\&+\{\mathfrak {N_1}x(t)+\bar{\beta }\mathfrak {{N}_2}K[(1-\bar{\alpha })e^{\mathfrak {N}_1(\Gamma ^{-1} (t)-t)} \psi _{\mathfrak {q}(t)} x(t-\varepsilon (t))\nonumber \\ &+\bar{\alpha } \mathcal {G}(x_\mathfrak {s} (t))]+\mathfrak {N_3}\omega (t)+\Im (t)\}^TPx(t)+x^T(t)\sum _{\mathfrak {s}=1}^{\mathfrak {a}}\pi _{\mathfrak {sm}}\Pi x(t). \end{aligned}$$

The analytical solution of the system (3) can be written as

16 $$\begin{aligned} x(t)=e^{\mathfrak {N}_1(t-\Gamma (t))}x(\Gamma (t))+\int _{\Gamma (t)}^{t}e^{\mathfrak {N}_1(t-\hbar ))}[\mathfrak {{N}_2}\mathfrak {u}(\Gamma (\hbar ))+\mathfrak {N_3}\omega (\hbar )+\Im (\hbar )]d\hbar . \end{aligned}$$

The compact form of (16) is obtained as

17 $$\begin{aligned} e^{\mathfrak {N}_1(t-\Gamma (t))}x(t-\varepsilon (t))=x(t)-(1-\bar{\alpha })\bar{\beta }\Upsilon _1(t)-\bar{\alpha }\bar{\beta }\Upsilon _2(t)-\Upsilon _3 (t)-\Upsilon _4 (t), \end{aligned}$$

where

$$\begin{aligned} \Upsilon _1(t)=\int _{\Gamma (t)}^{t} e^{\mathfrak {N}_1 (t-\hbar )}\mathfrak {N}_2 K e^{\mathfrak {N}_1\varepsilon (t)}x(\hbar -\varepsilon (t))d\hbar;& \Upsilon _3 (t)=\int _{\Gamma (t)}^{t} e^{\mathfrak {N}_1 (t-\hbar )}\mathfrak {N}_3\omega (\hbar ) d\hbar; \\\Upsilon _2(t)=\int _{\Gamma (t)}^{t} e^{\mathfrak {N}_1(t-\hbar )}\mathfrak {N}_2 K e^{\mathfrak {N}_1\varepsilon (t)}\mathcal {G}(x(\hbar )) d\hbar ;& \Upsilon _4(t)=\int _{\Gamma (t)}^{t} e^{\mathfrak {N}_1(t-\hbar )} \Im (\hbar ) d\hbar . \end{aligned}$$

From (15) and (17), we get

18 $$\begin{aligned} \mathbb {E}\{\mathcal {L}\dot{\mathcal {V}}_1 (t)\}=~&x^T(t)P\{\mathfrak {N_1}x(t)+\bar{\beta }\mathfrak {{N}_2}K[(1-\bar{\alpha }) \psi _{\mathfrak {q}(t)}\times (x(t)-(1-\bar{\alpha })\bar{\beta }\Upsilon _1(t)-\bar{\alpha }\bar{\beta }\Upsilon _2(t)-\Upsilon _3 (t)-\Upsilon _4 (t))+\bar{\alpha } \mathcal {G}(x_\mathfrak {s} (t))]\nonumber \\ &+\mathfrak {N_3}\omega (t)+\Im (t)\}+\{\mathfrak {N_1}x(t)+\bar{\beta }\mathfrak {{N}_2}K[(1-\bar{\alpha }) \psi _{\mathfrak {q}(t)}\times (x(t)-(1-\bar{\alpha })\bar{\beta }\Upsilon _1(t)-\bar{\alpha }\bar{\beta }\Upsilon _2(t)-\Upsilon _3 (t)-\Upsilon _4 (t))\nonumber \\ &+\bar{\alpha } \mathcal {G}(x_\mathfrak {s} (t))]+\mathfrak {N_3}\omega (t)+\Im (t)\}^TPx(t)+x^T(t)\sum _{\mathfrak {s}=1}^{\mathfrak {a}}\pi _{\mathfrak {sm}}\Pi x(t). \end{aligned}$$

According to Ref^39^., for any two vectors $\mathfrak {h}_1$ and $\mathfrak {h}_2$, we have

$$\begin{aligned}\pm (\mathfrak {h}_1^T \mathfrak {h}_2+\mathfrak {h}_2^T \mathfrak {h}_1)\le \mathfrak {h}_1^T\mathfrak {h}_1+\mathfrak {h}_2^T\mathfrak {h}_2.\end{aligned}$$

Using this result, we obtain

19 $$\begin{aligned} -\{x^T(t)[&PK\mathfrak {N}_2(1-\bar{\alpha })^2 \bar{\beta }^2\psi _{\mathfrak {q}(t)}]\Upsilon _1(t)+\Upsilon _1^T(t)[ \mathfrak {N}_2^T K^T P(1-\bar{\alpha })^2\bar{\beta }^2\psi _{\mathfrak {q}(t)}]x(t)\} \\&\le x^T(t)[(1-\bar{\alpha })^4\bar{\beta }^4 PK\mathfrak {N}_2 \psi _{\mathfrak {q}(t)}^2\mathfrak {N}_2^T K^T P]x(t)+\Upsilon _1^T(t)\Upsilon _1(t). \end{aligned}$$

Similarly, for the other terms

20 $$\begin{aligned} -\{x^T(t)[&PK\mathfrak {N}_2(1-\bar{\alpha })\bar{\alpha } \bar{\beta }^2\psi _{\mathfrak {q}(t)}]\Upsilon _2(t)+\Upsilon _2^T(t)[ \mathfrak {N}_2^T K^T P(1-\bar{\alpha })\bar{\alpha }\bar{\beta }^2\psi _{\mathfrak {q}(t)}]x(t)\}\\& \le x^T(t)[(1-\bar{\alpha })^2\bar{\alpha }^2\bar{\beta }^4 PK\mathfrak {N}_2 \psi _{\mathfrak {q}(t)}^2\mathfrak {N}_2^T K^T P]x(t)+\Upsilon _2^T(t)\Upsilon _2(t),\end{aligned}$$

21 $$\begin{aligned} -\{x^T(t)[&PK\mathfrak {N}_2(1-\bar{\alpha }) \bar{\beta }\psi _{\mathfrak {q}(t)}]\Upsilon _3(t)+\Upsilon _3^T(t)[ \mathfrak {N}_2^T K^T P(1-\bar{\alpha })\bar{\beta }\psi _{\mathfrak {q}(t)}]x(t)\}\\& \le x^T(t)[(1-\bar{\alpha })^2\bar{\beta }^2 PK\mathfrak {N}_2 \psi _{\mathfrak {q}(t)}^2\mathfrak {N}_2^T K^T P]x(t)+\Upsilon _3^T(t)\Upsilon _3(t),\end{aligned}$$

22 $$\begin{aligned} -\{x^T(t)[&PK\mathfrak {N}_2(1-\bar{\alpha }) \bar{\beta }\psi _{\mathfrak {q}(t)}]\Upsilon _4(t)+\Upsilon _4^T(t)[ \mathfrak {N}_2^T K^T P(1-\bar{\alpha })\bar{\beta }\psi _{\mathfrak {q}(t)}]x(t)\}\\&\le x^T(t)[(1-\bar{\alpha })^2\bar{\beta }^2 PK\mathfrak {N}_2 \psi _{\mathfrak {q}(t)}^2\mathfrak {N}_2^T K^T P]x(t)+\Upsilon _4^T(t)\Upsilon _4(t), \end{aligned}$$

23 $$\begin{aligned} -\{x^T(t)[&PK\mathfrak {N}_2\bar{\alpha } \bar{\beta }]\mathcal {G}(x_\mathfrak {s} (t))+\mathcal {G}^T(x_\mathfrak {s}(t))[\bar{\alpha }\bar{\beta } \mathfrak {N}_2^T K^T P]x(t)\}\le x^T(t)[\bar{\alpha }^2\bar{\beta }^2 PK\mathfrak {N}_2 \mathfrak {N}_2^T K^T P]x(t)+\mathcal {G}^T(x_\mathfrak {s} (t))\mathcal {G}(x_\mathfrak {s} (t)),\end{aligned}$$

24 $$\begin{aligned} \{x^T(t)&P\Im (t)+\Im ^T(t)Px(t)\}\le x^T(t)PPx(t)+\Im ^T(t)\Im (t). \end{aligned}$$

We know that $K=-\mathfrak {N}_2^TP$. Then, the first term of the right-hand side of (19)–(23) is derived as

25 $$\begin{aligned} \left\{ \begin{array}{ll} x^T(t)[(1-\bar{\alpha })^4\bar{\beta }^4 PK\mathfrak {N}_2 \psi _{\mathfrak {q}(t)}^2\mathfrak {N}_2^T K^T P]x(t)& \le P^2 (1-\bar{\alpha })^4\bar{\beta }^4 \psi _{\mathfrak {q}(t)}^2 x^T(t) \xi ^2 x(t),\\ x^T(t)[(1-\bar{\alpha })^2\bar{\alpha }^2\bar{\beta }^4 PK\mathfrak {N}_2 \psi _{\mathfrak {q}(t)}^2\mathfrak {N}_2^T K^T P]x(t)& \le P^2 (1-\bar{\alpha })^2\bar{\alpha }^2\bar{\beta }^4\psi _{\mathfrak {q}(t)}^2 x^T(t)\xi ^2 x(t),\\ x^T(t)[(1-\bar{\alpha })^2\bar{\beta }^2 PK\mathfrak {N}_2 \psi _{\mathfrak {q}(t)}^2\mathfrak {N}_2^T K^T P]x(t)& \le P^2 (1-\bar{\alpha })^2\bar{\beta }^2\psi _{\mathfrak {q}(t)}^2 x^T(t)\xi ^2 x(t),\\ x^T(t)[(1-\bar{\alpha })^2\bar{\beta }^2 PK\mathfrak {N}_2 \psi _{\mathfrak {q}(t)}^2\mathfrak {N}_2^T K^T P]x(t)& \le P^2 (1-\bar{\alpha })^2\bar{\beta }^2\psi _{\mathfrak {q}(t)}^2 x^T(t)\xi ^2 x(t),\\ x^T(t)[\bar{\alpha }^2\bar{\beta }^2 PK\mathfrak {N}_2 \mathfrak {N}_2^T K^T P]x(t)& \le P^2 \bar{\alpha }^2\bar{\beta }^2 x^T(t) \xi ^2 x(t). \end{array} \right. \end{aligned}$$

With the aid of Lemmas 1 and 2, and relations (11) and (12), the second term on the right-hand side of inequalities (19)–(22) is reformulated as follows:

26 $$\begin{aligned} \Upsilon _1^T(t)\Upsilon _1(t)&=\Bigg (\int _{\Gamma (t)}^{t} e^{\mathfrak {N}_1 (t-\hbar )}\mathfrak {N}_2 K e^{\mathfrak {N}_1\varepsilon (t)}x(\hbar -\varepsilon (t))d\hbar \Bigg )^T \Bigg (\int _{\Gamma (t)}^{t} e^{\mathfrak {N}_1 (t-\hbar )}\mathfrak {N}_2 K e^{\mathfrak {N}_1\varepsilon (t)}x(\hbar -\varepsilon (t))d\hbar \Bigg )\nonumber \\&\le \varepsilon _2~\xi ^2 e^{2\mu {\varepsilon }_2}\int _{t-\varepsilon (t)}^{t}x^T(\hbar -\varepsilon (t))x(\hbar -\varepsilon (t))d\hbar \end{aligned}$$

Using the transformation $\varrho =\Gamma (\hbar )$, we obtain $d\hbar =\Bigg (\frac{d}{d\hbar }\Gamma (\hbar )\Bigg |_{\hbar =\Gamma ^{-1}(\varrho )}\Bigg )^{-1}d\varrho .$ Then,

27 $$\begin{aligned} \Vert \Upsilon _1(t)\Vert ^2&\le \varepsilon _2~\xi ^2 e^{2\mu {\varepsilon }_2}\int _{\Gamma (\Gamma (t))}^{\Gamma (t)}x^T(\varrho )x(\varrho )\Bigg (\frac{d}{d\hbar }\Gamma (\hbar )\Bigg |_{\hbar =\Gamma ^{-1}(\varrho )}\Bigg )^{-1}d\varrho \nonumber \\&\le \varepsilon _2~\xi ^2 e^{2\mu {\varepsilon }_2}\eta ^{-1}\int _{\Gamma (\Gamma (t))}^{\Gamma (t)}x^T(\varrho )x(\varrho )d\varrho \nonumber \\&\le \varepsilon _2~\xi ^2 e^{2\mu {\varepsilon }_2}\eta ^{-1}\int ^{t}_{t-2\hbar }x^T(\varrho )x(\varrho )d\varrho . \end{aligned}$$

Similarly,

28 $$\begin{aligned} ||\Upsilon _2(t)||^2&\le \varepsilon _2~\xi ^2 e^{2\mu {\varepsilon }_2}\eta ^{-1}\int ^{t}_{t-2\hbar }\mathcal {G}^T(x_\mathfrak {s}(\varrho ))\mathcal {G}(x_\mathfrak {s}(\varrho ))d\varrho ,\end{aligned}$$

29 $$\begin{aligned} ||\Upsilon _3(t)||^2&\le \varepsilon _2 e^{\mu {\varepsilon }_2}\eta ^{-1}\int ^{t}_{t-2\hbar }\omega ^T(\varrho )\bar{\mathcal {N}_3}\omega (\varrho )d\varrho ,\end{aligned}$$

30 $$\begin{aligned} ||\Upsilon _4(t)||^2&\le \varepsilon _2 e^{\mu {\varepsilon }_2}\eta ^{-1}\int ^{t}_{t-2\hbar }\mathfrak {F}^T(\varrho )\mathfrak {F}(\varrho )d\varrho . \end{aligned}$$

So the Total LKFs are

$$\begin{aligned}{{V}}^{1} (t)=\sum _{i=1}^{5}{\mathcal {V}}_i(t),\end{aligned}$$

where

31 $$\begin{aligned} {\mathcal {V}}_2(t)=&\int ^{t}_{t-\varepsilon (t)} x^T(\varrho )Q_1 x(\varrho ) d\varrho +\int ^{t}_{t-\varepsilon _2} x^T(\varrho )Q_2 x(\varrho ) d\varrho , \end{aligned}$$

32 $$\begin{aligned} {\mathcal {V}}_3(t)=&\varepsilon _2~\xi ^2 e^{2\mu {\varepsilon }_2}\eta ^{-1}\int _{0}^{2\hbar }\int ^{t}_{t-\hbar }x^T(\varrho)x(\varrho )d\varrho d\hbar , \end{aligned}$$

33 $$\begin{aligned} {\mathcal {V}}_4(t)=&\varepsilon _2~\xi ^2 e^{2\mu {\varepsilon }_2}\eta ^{-1}\int _{0}^{2\hbar }\int ^{t}_{t-\hbar }\mathcal {G}^T(x_\mathfrak {s}(\varrho ))\mathcal {G}(x_\mathfrak {s}(\varrho ))d\varrho d\hbar , \end{aligned}$$

34 $$\begin{aligned} {\mathcal {V}}_5(t)=&\varepsilon _2 e^{\mu {\varepsilon }_2}\eta ^{-1}\int _{0}^{2\hbar }\int ^{t}_{t-\hbar }\omega ^T(\varrho )\omega (\varrho )d\varrho d\hbar +\varepsilon _2 e^{\mu {\varepsilon }_2}\eta ^{-1}\int _{0}^{2\hbar }\int ^{t}_{t-\hbar }\Im ^T(\varrho )\Im (\varrho )d\varrho d\hbar . \end{aligned}$$

Then the corresponding time derivatives are given by

35 $$\begin{aligned} \mathbb {E}\{\mathcal {L}\dot{\mathcal {V}}_2 (t)\}=x^T(t)[Q_1+Q_2]x(t)-x^T(t-\varepsilon _2)Q_2x(t-\varepsilon _2)-x^T(t-\varepsilon(t))Q_1(1-\delta)x(t-\varepsilon(t)),\end{aligned}$$

36 $$\begin{aligned} \mathbb {E}\{\mathcal {L}\dot{\mathcal {V}}_3 (t)\}\le&~x^T(t) 2\varepsilon _2^2~\xi ^2 e^{2\mu {\varepsilon }_2}\eta ^{-1}x(t)-\varepsilon _2~\xi ^2 e^{2\mu {\varepsilon }_2}\eta ^{-1}\int _{t-2\hbar }^{t}x^T(\varrho )x(\varrho )d\varrho , \end{aligned}$$

37 $$\begin{aligned} \mathbb {E}\{\mathcal {L}\dot{\mathcal {V}}_4 (t)\}\le&~2\varepsilon _2^2~\xi ^2 e^{2\mu {\varepsilon }_2}\eta ^{-1}\mathcal {G}^T(x_\mathfrak {s}(t))\mathcal {G}(x_\mathfrak {s}(t))-\varepsilon _2~\xi ^2 e^{2\mu {\varepsilon }_2}\eta ^{-1}\int _{t-2\hbar }^{t}\mathcal {G}^T(x_\mathfrak {s}(\varrho ))\mathcal {G}(x_\mathfrak {s}(\varrho ))d\varrho , \end{aligned}$$

38 $$\begin{aligned} \mathbb {E}\{\mathcal {L}\dot{\mathcal {V}}_5 (t)\}\le&~2\varepsilon _2^2e^{\mu {\varepsilon }_2}\eta ^{-1}\omega ^T(t)\bar{\mathcal {N}_3}\omega (t)+2\varepsilon _2^2e^{\mu {\varepsilon }_2}\eta ^{-1}\Im ^T (t)\Im (t)-\varepsilon _2 e^{\mu {\varepsilon }_2}\eta ^{-1}\int _{t-2\hbar }^{t}\omega ^T(\varrho )\bar{\mathcal {N}_3}\omega (\varrho )d\varrho -\varepsilon _2 e^{\mu {\varepsilon }_2}\eta ^{-1}\int _{t-2\hbar }^{t}\Im ^T (\varrho )\Im (\varrho )d\varrho . \end{aligned}$$

From system (10) it is observed that

39 $$\begin{aligned} 0=2&zP[-x(t-\varepsilon (t))+\dot{x}(t)-\omega (t)]\times [- \dot{x}(t)+\mathfrak {N_1}x(t)+\bar{\beta }\mathfrak {{N}_2}K[(1-\bar{\alpha })\times e^{\mathfrak {N}_1(\Gamma ^{-1} (t)-t)} \psi _{\mathfrak {q}(t)} x(t-\varepsilon (t))\\&+\bar{\alpha } \mathcal {G}(x_\mathfrak {s} (t))]+\mathfrak {N_3}\omega (t)+\Im (t)]. \end{aligned}$$

Combining (14)–(39), yields

40 $$\begin{aligned} \mathbb {E}\{\mathcal {L}\dot{{V}}^1 (t)-\mathfrak {J}(t)\}\le \Xi (t)\Theta \Xi ^T(t),\end{aligned}$$

where $$\Xi ^T(t)=[x^T(t)~x^T(t-\varepsilon (t))~x^T(t-\varepsilon _2)~\mathcal {G}^T(x_\mathfrak {s}(t))~\dot{x}^T(t)~w^T(t)]$$.

**Case 2:** Consider the occurrence of DoSA, i.e. $\bar{\beta }= 0.$ The corresponding LKFs are selected as in (15) and (32). Thus,

$$\begin{aligned}{V}^{2} (t)=\sum _{i=1}^{2}{\mathcal {V}}_i (t).\end{aligned}$$

Proceeding similarly to Theorem 1, we arrive at the following expression

41 $$\begin{aligned} \mathbb {E}\{\mathcal {L}\dot{{V}}^2 (t)-\mathfrak {J}(t)\}\le \tilde{\Xi }(t)\tilde{\Theta }\tilde{\Xi }^T(t),\end{aligned}$$

where $$\tilde{\Xi }^T(t)=[x^T(t)~x^T(t-\varepsilon (t))~x^T(t-\varepsilon _2)~w^T(t)]$$.

Combining (40) and (41), we have

42 $$\begin{aligned} \mathbb {E}\{\mathcal {L}\dot{{V}}(t)\}\le \mathbb {E}\{\mathfrak {J}(t)\},\end{aligned}$$

where $\dot{V}(t)$ takes different forms over the domains $\mathfrak {D}_{1,n}$ and $\mathfrak {D}_{2,n}$ as given below

$$\begin{aligned} \dot{V }(t)=\left\{ \begin{array}{ll} \dot{V}^1 (t),~t \in \mathfrak {D}_{1,n} \\ \dot{V}^2 (t),~t \in \mathfrak {D}_{2,n}. \end{array} \right. \end{aligned}$$

Integrating (42) from 0 to $\bar{T}$, then we have

43 $$\begin{aligned} x^T(t)Px(t)\le \mathbb {E}\{\mathcal {V}(t)-\mathcal {V}(0)\}\le \mathbb {E}\left\{ \int _{0}^{\bar{T}}\mathfrak {J}(t)dt\right\} .\end{aligned}$$

From Definition 1, $H_{\infty }$, passivity, and $(\mathcal {Q},\mathcal {S}, \mathcal {R})$-dissipativity hold for $\Lambda _4 =0$, while $L_2-L_{\infty }$ performance applies when $\Lambda _4>0$. Considering $\Lambda _4 =0$, we obtain

$$\begin{aligned}\mathbb {E}\left\{ \int _{0}^{\bar{T}}\mathfrak {J}(t)dt\right\} \ge 0.\end{aligned}$$

Accordingly, Definition 1 holds when $\Lambda _4=0.$ Now, consider $\Lambda _4>0$ with $\Lambda _1=\Lambda _2=0$ and $\Lambda _3>0$ as in Assumption 3. For any $0\le t\le \bar{T}$ and considering (43), we can obtain $\mathbb {E}\{\int _{0}^{\bar{T}}\mathfrak {J}(t)dt\}\ge \mathbb {E}\{\int _{0}^{{T}}\mathfrak {J}(t)dt\}\ge \mathbb {E}\{x^T(t)Px(t)\}>0.$ Hence, it follows that:

$$\begin{aligned}\mathbb {E}\{y^T(t)\Lambda _4 y(t)\}=\mathbb {E}\{x^T(t)\mathfrak {N}_4^T \Lambda _4 \mathfrak {N}_4 x(t)\}\le \mathbb {E}\{x^T(t)Px(t)\le \mathbb {E}\left\{ \int _{0}^{\bar{T}}\mathfrak {J}(t)dt\right\} .\end{aligned}$$

For both $\Lambda _4=0$ and $\Lambda _4>0$, system (10) is asymptotically stable and satisfies the EDP with respect to $\omega (t)$. Hence, the proof is concluded.$\square$

### Remark 6

Considering the uncertain parameters $\Delta N_{\varpi } (t)=0~(\varpi =1,2,3,4)$ with the sleeping period of DoSA $(i.e. \beta =1).$ Then (10) can be written as

44 $$\begin{aligned} \left\{ \begin{array}{ll} \dot{x}(t)={N_1}x(t)+{N}_2 K[(1-\bar{\alpha })e^{{N}_1(\Gamma ^{-1} (t)-t)} \psi _{\mathfrak {q}(t)}x(t-\varepsilon (t))\bar{\alpha } \mathcal {G}(x_\mathfrak {s}(t))]+{N_3}\omega (t)+\Im (t),\\ y(t)={N}_4 x(t). \end{array} \right. \end{aligned}$$

Correspondingly, the above system (44) is MSAS, which is carried out via the following Corollary.

### Corollary 1

*Given scalars* $\varepsilon _2,~\xi ,~\mu ,~{z}>0$ *and matrices* $\mathfrak {S}>0,~\Lambda _1~\Lambda _2,~\Lambda _3,~\Lambda _4.$ *System* (44) *is MSAS with* $\mathcal {X}=P^{-1}$ *and the controller gain* ${K}=-{N}_2^TP$, *if positive symmetric matrices* $P,Q_1,Q_2$ *exists and meets the following conditions*:

45 $$\begin{aligned} {N}_2 {N}_2^T\le \xi \mathcal {X} \end{aligned}$$

46 $$\begin{aligned} \Bigg ({N}_1-\frac{1}{2}\mu I\Bigg )^T+\Bigg ({N}_1-\frac{1}{2}\mu I\Bigg )<0 \end{aligned}$$

47 $$\begin{aligned}~[\Omega ]_{6\times 6}<0 \end{aligned}$$

where 

$$\Omega _{11}=P{N}_1+{N}_1^TP-2(1-\bar{\alpha })\bar{\beta }P^2 {N}_2^2 \psi _{\mathfrak {q}(t)}+\Pi +(1-\bar{\alpha })^4\bar{\beta }^4 \xi ^2 P^2 \psi ^2_{\mathfrak {q}(t)}+(1-\bar{\alpha })^2\bar{\alpha }^2\bar{\beta }^4 \xi ^2 P^2 \psi ^2_{\mathfrak {q} (t)}+2(1-\bar{\alpha })^2\bar{\beta }^2 \xi ^2 P^2 \psi ^2_{\mathfrak {q} (t)}$$ $$+\bar{\alpha }^2\bar{\beta }^2 {N}_2^4 P^4+5\mathfrak {S}^T\mathfrak {S}+P^2+4\mathcal {N}_1^2+Q_1+Q_2+2\varepsilon _2^2\xi ^2 e^{2\mu \varepsilon _2}\eta ^{-1}+2\varepsilon _2^2\xi ^2 e^{2\mu \varepsilon _2}\eta ^{-1}\mathfrak {S}\mathfrak {S}^T+2\varepsilon _2^2 e^{\mu \varepsilon _2}\eta ^{-1}\mathcal {N}_1^2-{N}_4\Lambda _1{N}_4^T$$ $$+P-{N}_4\Lambda _4 {N}_4^T, \Omega _{13}=2zP {N}_1,\Omega _{15}=2zP {N}_1,\Omega _{16}=P {N}_3+ {N}_3^T P+8\mathcal {N}_1\mathcal {N}_3+4\varepsilon _2^2 e^{\mu \varepsilon _2} \eta ^{-1}\mathcal {N}_1\mathcal {N}_3-2zP {N}_1- {N}_4\Lambda _2,$$ $$\Omega _{22}=-Q_1(1-\delta ),\Omega _{33}=-Q_2-2zP^2 (1-\bar{\alpha })\bar{\beta } {N}_2^2 e^{ {N}_1 \varepsilon _2}\psi _{\mathfrak {q} (t)}+z^2 P^4\bar{\alpha }^2\bar{\beta }^2 {N}_2^4+z^2P^2,\Omega _{35}=-2zP-2zP^2 (1-\bar{\alpha })\bar{\beta } {N}_2^2 e^{ {N}_1 \varepsilon _2}\psi _{\mathfrak {q} (t)}$$ $$+z^2 P^4\bar{\alpha }^2\bar{\beta }^2 {N}_2^4,\Omega _{36}=2zP {N}_3+2zP^2 (1-\bar{\alpha })\bar{\beta } {N}_2^2 e^{ {N}_1 \varepsilon _2}\psi _{\mathfrak {q}(t)},$$ $$\Omega _{44}=-I,\Omega _{55}=-2zP+z^2 P^2,\Omega _{56}=2zP {N}_3+2z P,\Omega _{66}=4\mathcal {N}_3^2+2\varepsilon _2^2$$ $$e^{\mu \varepsilon _2}\eta ^{-1} {N}_3^2+2\varepsilon _2^2 e^{\mu \varepsilon _2}\eta ^{-1}\bar{N}_3-2zP {N}_3+z^2 P^4\bar{\alpha }^2\bar{\beta }^2 {N}_2^4+z^2P^2-\Lambda _3.$$

### Proof

It is clear that Theorem 1 is true. Then (45) and (46) are easily found to be identical to (11) and (12), respectively. By using (13), we can confirm that $\Theta <0$ is equivalent to $\Omega <0,$ with $\mathcal {X}=P^{-1}$. The system (44) is therefore MSAS, according to Definition 1. Hence, the proof is concluded. $\square$

## Numerical example

To validate the results, two computational examples are provided in this section.

### Example 1

The matrix variables of (10) are considered to be

$$\begin{aligned}&{N}_1=\left[ \begin{array}{cc} -2 & 0 \\ 0 & -2 \end{array} \right] ,~\Delta {N}_1=\left[ \begin{array}{cc} -2& 0 \\ 0 & -2 \end{array} \right] \mathcal {Z}(t) \left[ \begin{array}{cc} -2 & 0 \\ 0 & -0.68 \end{array} \right] ,~{N}_2=\pi *\left[ \begin{array}{cc} 0.1 & 0 \\ 0 & 0.2 \end{array} \right] ,\Delta {N}_2=\left[ \begin{array}{cc} 0.01 & 1 \\ 1 & 0.5 \end{array} \right] \mathcal {Z}(t) \left[ \begin{array}{cc} 0.5 & 0 \\ 0 & 0.68 \end{array} \right] ,\\&{N}_3=\left[ \begin{array}{cc} 0.01 & 0 \\ 0 & 0.01\\ \end{array} \right] ,~\Delta {N}_3=\left[ \begin{array}{cc} 0.1 & 0 \\ 0 & 0.1 \end{array} \right] \mathcal {Z}(t) \left[ \begin{array}{cc} 0.1 & 0 \\ 0 & 0.1 \end{array} \right] ,{N}_4 =\left[ \begin{array}{cc} 0.05 & 0\\ 0 & 0.02\\ \end{array} \right] ,~\Delta {N}_4=\left[ \begin{array}{cc} 0.12 & 0 \\ 0 & 0.1 \end{array} \right] \mathcal {Z}(t) \left[ \begin{array}{cc} 0.1 & 0 \\ 0 & 0.18 \end{array} \right],\\ &\mathcal {G}=\left[ \begin{array}{cc} 1 & 0 \\ 0 & 1 \end{array} \right] ,~\Pi =\left[ \begin{array}{cc} 0.8 & 0.2 \\ 0.9 & 0.1 \end{array} \right] ,and~\mathcal {Z}(t)= sin (\pi t). \end{aligned}$$

Furthermore, the matrix associated with (2) are

$$\begin{aligned} \mathcal {N}_1=\left[ \begin{array}{cc} -0.3 & 0 \\ 0 & -0.3 \end{array} \right] ,~\mathcal {N}_3=\left[ \begin{array}{cc} -1 & 0 \\ 0 & -1 \end{array} \right] . \end{aligned}$$

**Case 1: Without DoSA**

Let $\varepsilon _2=0.001,~\eta =0.04,~\xi =0.2,~\mu =0.001,\delta =0.9,~z=6.0600e-05,~\bar{\alpha }=0.1,$ and $~\bar{\beta }=0.1$. We utilize MATLAB’s LMI Toolbox to solve the LMI formulated in Theorem 1, and the corresponding optimal performance indices for $H_\infty$ performance, $L_2-L_\infty$ performance, passivity performance, and $(\mathcal {Q}, \mathcal {S}, \mathcal {R})$– dissipative performance are simultaneously obtained.

**Fig. 2:**
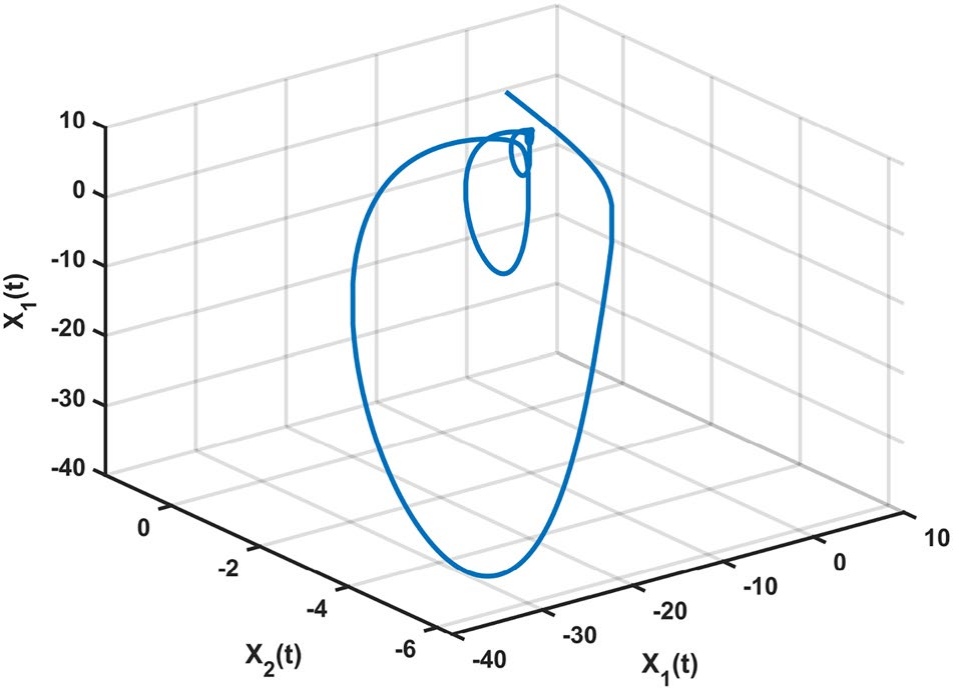
Chaotic behavior of system (10).

**Fig. 3:**
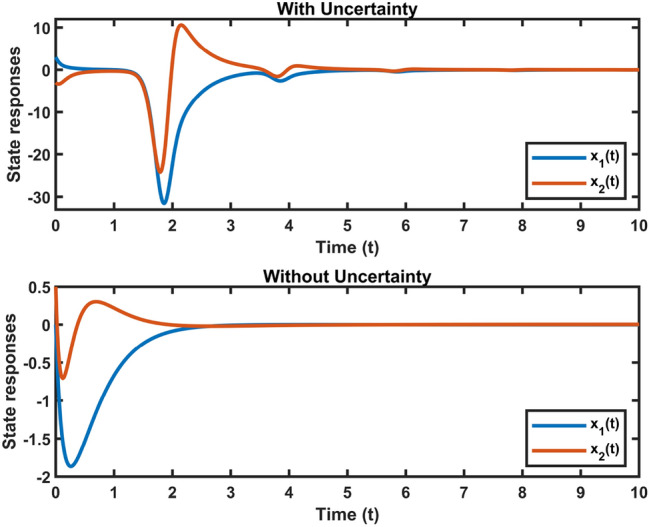
Dynamic behavior of system states in Case 1.

**Fig. 4:**
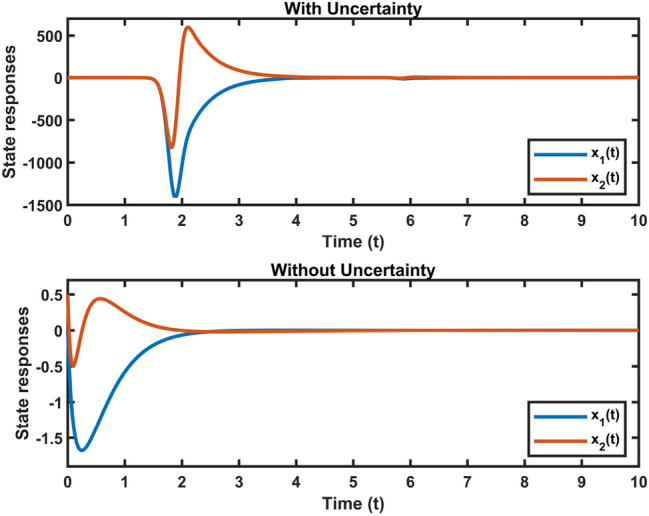
Dynamic behavior of system states in Case 2.

**Fig. 5:**
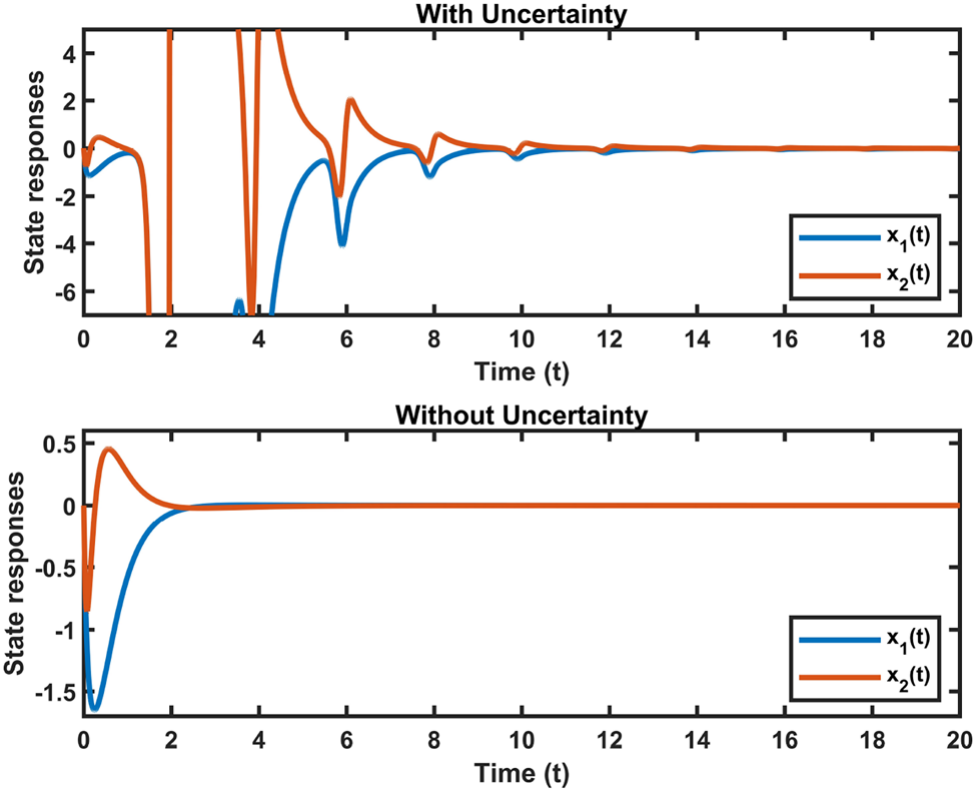
Dynamic behavior of system states in Case 3.

**Fig. 6:**
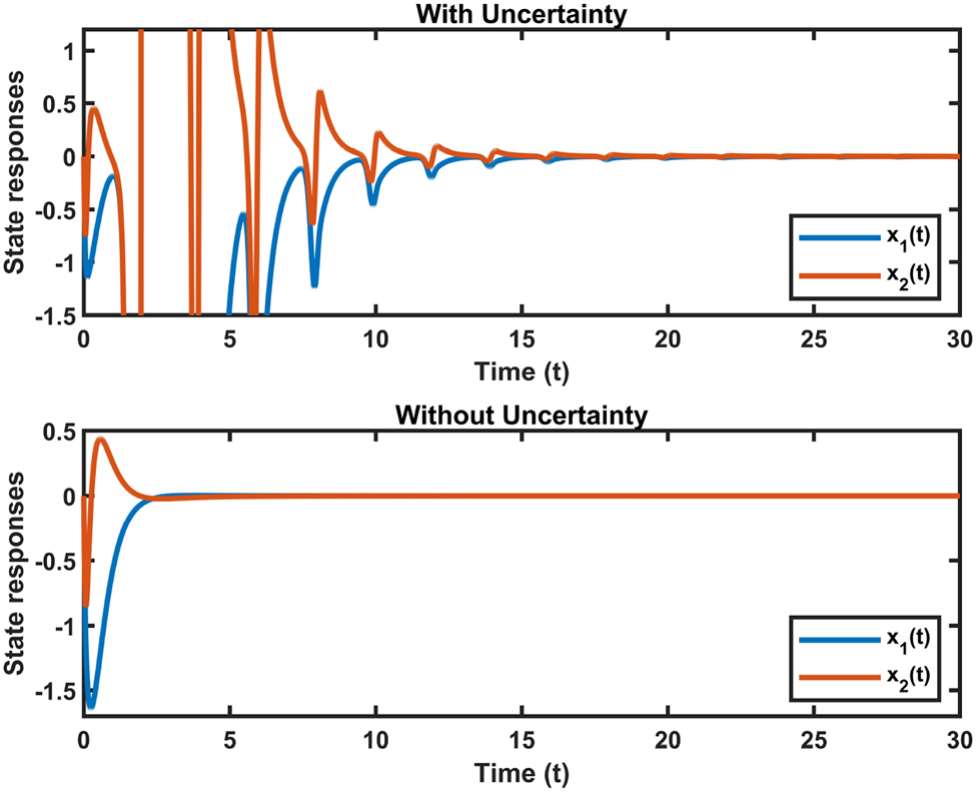
Dynamic behavior of system states in Case 4.

$\mathbf {C_1}.~H_{\infty }$ performance: Let $\Lambda _1=-I,\Lambda _2=0,\Lambda _3=\nu ^2I,\Lambda _4=0.$ Also, one may derive the performance index as $\nu =2.17$. Then, the corresponding controller gain is

$$\begin{aligned}K=\left[ \begin{array}{cc} -52.6187& -14.9823\\ -29.9647& -90.6515 \end{array} \right] .\end{aligned}$$

$\mathbf {C_2}.~L_2-L_{\infty }$ performance: Let $\Lambda _1=0,\Lambda _2=0,\Lambda _3=\nu ^2I,\Lambda _4=I.$ Also, one may derive the performance index as $\nu =2.3247$. Then, the controller gain is derived as

$$\begin{aligned}K=\left[ \begin{array}{cc} -86.2462& -18.3122\\ -36.6244& -131.9170 \end{array} \right] .\end{aligned}$$

$\mathbf {C_3}$. Passivity performance: Let $\Lambda _1=0,\Lambda _2=I,\Lambda _3=\nu ^2I,\Lambda _4=0.$ Also, one may derive the performance index as $\nu =2.3247$. Then, the LMI solution yields the controller gain as

$$\begin{aligned}K=\left[ \begin{array}{cc} -85.4768& -18.9143\\ -37.8286& -131.0171 \end{array} \right] .\end{aligned}$$

$\mathbf {C_4}$. Dissipative performance: Let $\Lambda _1=\mathcal {Q},\Lambda _2=\mathcal {S},\Lambda _3=\mathcal {R}-\nu ^2I,\Lambda _4=0.$ Also, one may derive the performance index as $\nu =-5$. The matrices $\mathcal {Q},\mathcal {S}$ and $\mathcal {R}$ are given by $$\mathcal {Q}=I,\mathcal {S}=I,\mathcal {R}=\left[ \begin{array}{cc} 0.1 & 0.01 \\ 0.01 & 0.1 \end{array} \right] .$$ Then, the corresponding controller gain is obtained as

$$\begin{aligned}K=\left[ \begin{array}{cc} -83.6166& -22.0134\\ -44.0269& -124.4191 \end{array} \right] .\end{aligned}$$

**Case 2: With DoSA**

Let the parameter are considering as in case 1 with $\bar{\beta }=0.$ Then the system (10) is found to be MSAS by using the $\hbox {MATLAB}^{\circledR }$ LMI tool box. Accordingly, the gain matrices corresponding to the four cases $\mathbf {C_1-C_4}$ are obtained as follows:

$$\begin{aligned}K=\left[ \begin{array}{cc} -0.6490& -0.0777\\ -0.1555& -1.3755 \end{array} \right],~K=\left[ \begin{array}{cc} -0.7550& -0.0915\\ -0.1830& -1.5973 \end{array} \right],~K=\left[ \begin{array}{cc} -0.7532& -0.0963\\ -0.1925& -1.5698 \end{array} \right],~K=\left[ \begin{array}{cc} -0.7184& -0.0912\\ -0.1824& -1.5006 \end{array} \right] .\end{aligned}$$

**Table 1:** Upper bound of $\varepsilon _2$ with different value of attack parameters.

Parameters	$\mathbf {C_1}$	$\mathbf {C_2}$	$\mathbf {C_3}$	$\mathbf {C_4}$
$\bar{\alpha }=0.5, \bar{\beta }=1$	0.0763	0.1122	0.1124	0.0988
$\bar{\alpha }=0.1, \bar{\beta }=0.1$	0.0755	0.1116	0.1118	0.0982
$\bar{\alpha }=0.5, \bar{\beta }=0.5$	0.0759	0.1119	0.1121	0.0985
$\bar{\alpha }=1, \bar{\beta }=1$	0.0747	0.1110	0.1112	0.0975
$\bar{\alpha }=0.5,\bar{\beta }=0$	0.0742	0.1114	0.1116	0.0979

**Table 2:** Upper bound of $\varepsilon _2$.

Methods	In Ref^40^. Example 1	In Ref^40^. Example 2	$\mathbf {C_1}$	$\mathbf {C_2}$	$\mathbf {C_3}$	$\mathbf {C_4}$
$\varepsilon _2$	0.0602	0.025	0.0759	0.1119	0.1121	0.0985

**Table 3:** Controller gains at $\Delta N_{\varpi } (t)=0~(\varpi =1,2,3,4).$.

Case	$\nu$	Gain *K* with DoSA	Gain *K* without DoSA
$\mathbf {C_1}$	2.17	$$\left[ \begin{array}{cc} -52.6187& -14.9823\\ -29.9647& -90.6515 \end{array}\right]$$	$$\left[ \begin{array}{cc} -0.6490& -0.0777\\ -0.1555& -1.3755 \end{array}\right]$$
$\mathbf {C_2}$	2.3247	$$\left[ \begin{array}{cc} -86.2462& -18.3122\\ -36.6244& -131.9170 \end{array}\right]$$	$$\left[ \begin{array}{cc} -0.7550& -0.0915\\ -0.1830& -1.5973 \end{array}\right]$$
$\mathbf {C_3}$	2.3247	$$\left[ \begin{array}{cc} -85.4768& -18.9143\\ -37.8286& -131.0171 \end{array}\right]$$	$$\left[ \begin{array}{cc} -0.7532& -0.0963\\ -0.1925& -1.5698 \end{array}\right]$$
$\mathbf {C_4}$	-5	$$\left[ \begin{array}{cc} -83.6166& -22.0134\\ -44.0269& -124.4191 \end{array}\right]$$	$$\left[ \begin{array}{cc} -0.7184& -0.0912\\ -0.1824& -1.5006 \end{array}\right]$$

**Fig. 7:**
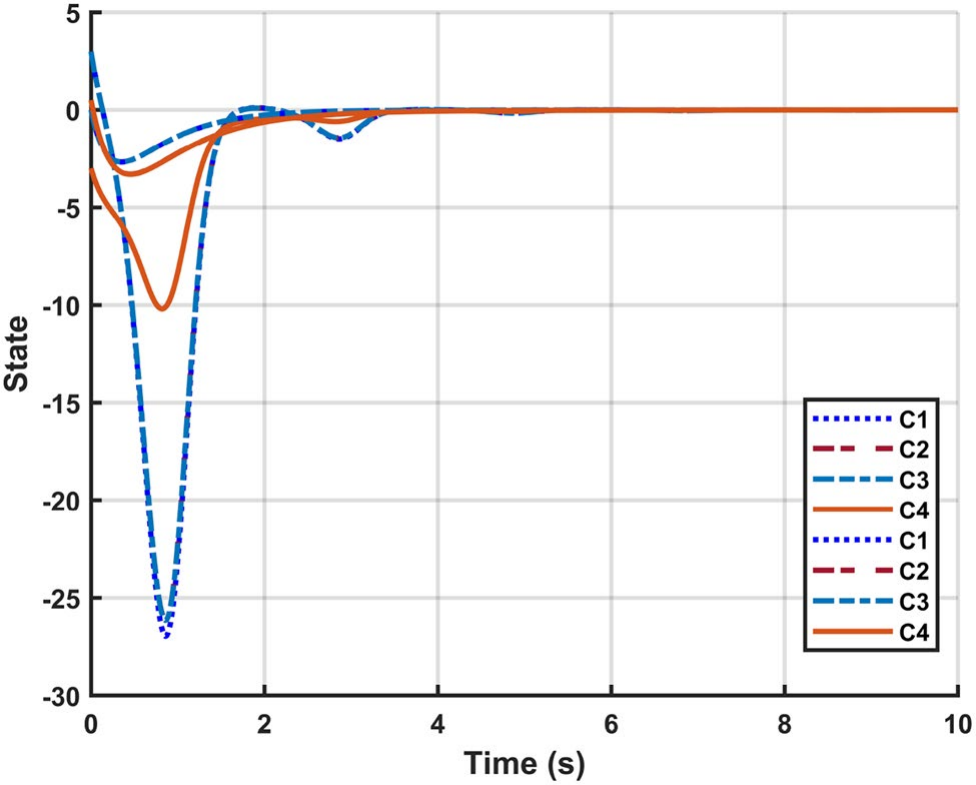
System response with DoSA.

**Fig. 8:**
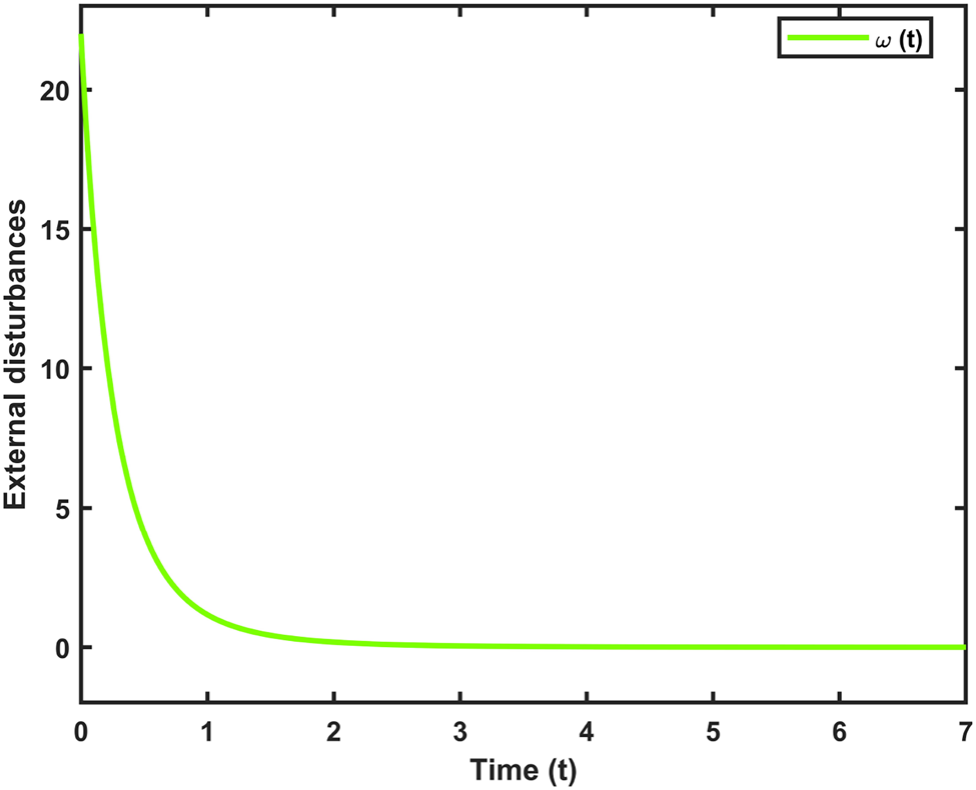
The external input disturbance.

**Fig. 9:**
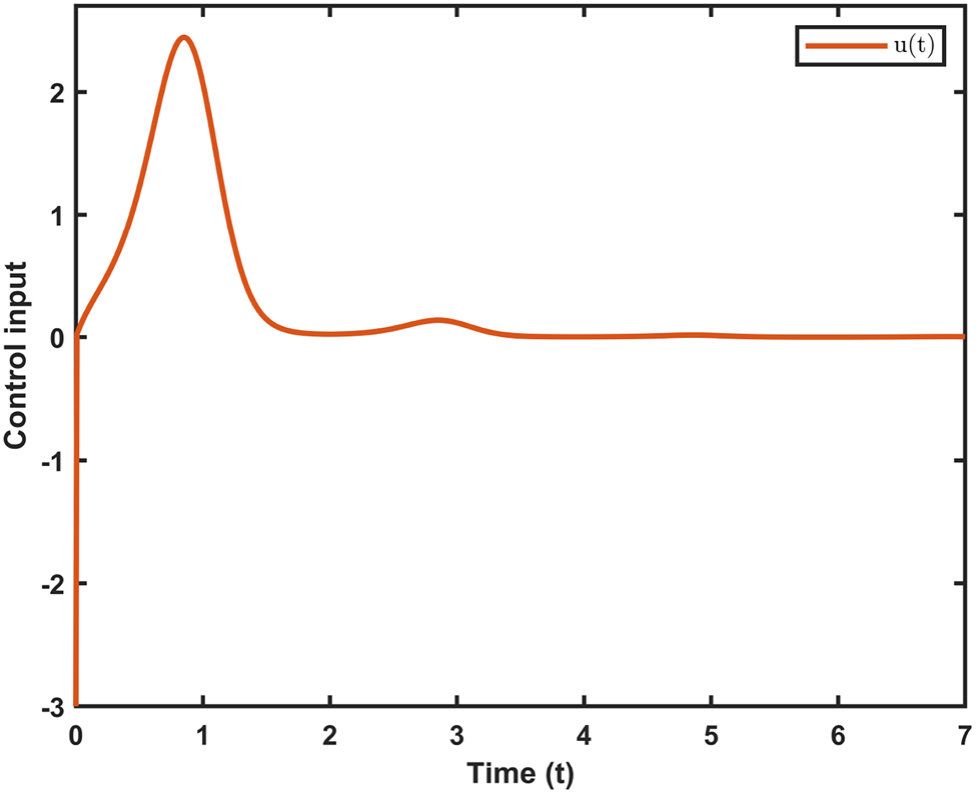
Control input $\mathfrak {u}(t)$ of system (10).

**Fig. 10:**
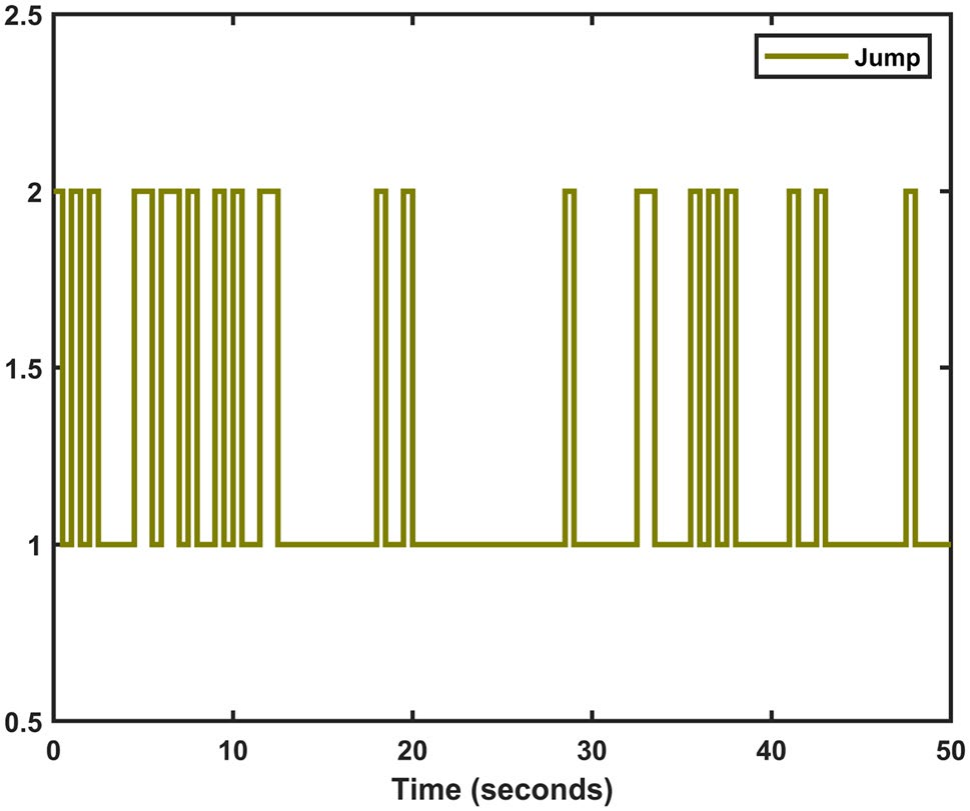
Jumping modes for Markov process.

Let $\omega (t)=[\frac{22}{(1+0.8*t)^{5}};\frac{23}{(1+0.5*t)^{5}}],~\mathcal {G}(x_{\mathfrak {s}}(t))=[-tanh(0.01x_2(t)); -tanh(0.01x_1(t))],$ are the external disturbance and nonlinear attack functions, respectively. Chaotic behavior of system (10) is shown in Fig. 2. Figures 3, 4, 5 and 6 depict the system’s convergence behavior across the four cases with and without uncertainty under the sleeping period of DoSA. Correspondingly, the state trajectory results without DoSA are presented in Fig. 7. The external and control input dynamics are depicted in Figs. 8 and 9, respectively. Figure 10 shows a Markov model of sensor distortion for the given system. For system (10), Table 1 shows identical upper bounds of $\varepsilon _2$ with and without uncertainty for various $\bar{\alpha }$ and $\bar{\beta }$. Table 2 shows that the proposed method provides a larger upper bound of $\varepsilon _2$ and achieves superior performance compared to the existing approach. Example 1 considers uncertainty, and the controller gains without uncertainty are listed in Table 3. Clearly, the CNNCS (11) achieves MSAS with EDP under sensor distortion and cyber attacks via TPC.

### Remark 7

In a similar manner, when $\Lambda _1=-\kappa I,\Lambda _2=(1-\kappa )I,\Lambda _3=[(\nu ^2 - \nu )\kappa + \nu ]I,\Lambda _4=0,$ with $0 \le \kappa \le 1$, the EDP reduces to a mixed $H_\infty$/passivity performance. The corresponding performance index can be obtained similarly using Theorem 1.

### Example 2

Consider the system (44) with the same parameters as in Example 1. In addition, $\Delta N_{\varpi } (t)=0~(\varpi =1,2,3,4).$ The LMI presented in Corollary 1 is solved by using LMI $\hbox {MATLAB}^{\circledR }$ Tool box, and the corresponding optimal performance indices for $H_\infty$, $L_2$–$L_\infty$, passivity, and $(\mathcal {Q}, \mathcal {S}, \mathcal {R})$-dissipativity are simultaneously obtained. This yields the follwoing gain matrices for the four cases $\mathbf {C_1-C_4}$:

$$\begin{aligned}K=\left[ \begin{array}{cc} -0.6452& -0.0760\\ -0.1520& -1.3736 \end{array} \right],~K=\left[ \begin{array}{cc} -0.7506& -0.0895\\ -0.1789& -1.5952 \end{array} \right],~K=\left[ \begin{array}{cc} -0.7496& -0.0946\\ -0.1892& -1.5691 \end{array} \right],~K=\left[ \begin{array}{cc} -85.6808& -11.6411\\ -23.2821& -181.4148 \end{array} \right] .\end{aligned}$$

Thus, the system (44) is found to be MSAS.

### Remark 8

The computational complexity of the proposed algorithm primarily arises from solving the LMIs derived from the LKF conditions. As linear, convex quadratic, and control-related constraints, such as Lyapunov and Riccati inequalities, can be represented as LMIs, they serve as an efficient tool for optimization and control design. In this work, Theorem 1 establishes sufficient LMI-based conditions for the asymptotic stabilization of CNNCSs under EDP and TPC. The computational load mainly depends on the number of decision variables and the size of the LMIs. In the numerical example, feasibility was verified under four cases of EDP, and the desired controller gain was obtained using MATLAB’s LMI Toolbox, which effectively reduces computational burden.

## Conclusion

An extended dissipative TPC strategy is proposed for delayed CNNCSs subject to uncertainty, sensor distortion, and cyber attacks. The EDP is addressed in terms of $H_{\infty }, L_2-L_{\infty },$ passivity and $(\mathcal {Q},\mathcal {S},\mathcal {R})$-dissipativity. Transmitting the sampled signal over the network introduces vulnerability to cyber attacks, which are represented by a Bernoulli distribution. Simultaneously, sensor distortion is represented using a Markov jump model. By applying LST, sufficient conditions are derived to guarantee system stability along with extended dissipativity, and the corresponding controller gain can be systematically computed. Two numerical examples are provided to validate the proposed approach. Also, the results demonstrate a significant reduction in conservatism compared to existing approaches. Future research can be focused on diverse attack mechanisms in network communication, on extending the analysis to more complex dynamic networks-especially T-S fuzzy-based NCSs-and on practical implementation of the proposed method to further validate its effectiveness and applicability.

## Data Availability

No datasets were generated or analysed during the current study
